# Smallest Detectable Change of the Knowledge and Attitudes of Pain in a High-Presenteeism Population: Female Teleworkers with Chronic Neck/Shoulder Stiffness

**DOI:** 10.7759/cureus.109453

**Published:** 2026-05-22

**Authors:** Hiroshi Takasaki

**Affiliations:** 1 Department of Physical Therapy, Saitama Prefectural University, Koshigaya, JPN

**Keywords:** interpretability, measurement error, outcome measurement, pain neuroscience education (pne), test-retest reliability

## Abstract

Background

Pain Science Education (PSE) aims to improve patients’ understanding of pain based on modern pain science, empowering informed care choices and supporting better health outcomes and has demonstrated effectiveness in chronic musculoskeletal conditions. The Knowledge and Attitudes of Pain (KNAP) questionnaire was developed to assess the knowledge and attitudes about pain science in physical therapy students undergoing PSE. In 2022, a Japanese-language version was created that is comprehensible to university students without specialized knowledge of pain. The KNAP is expected to assess changes in patients’ knowledge of pain science and attitudes toward pain among those receiving PSE, but its measurement error in patient populations remains unclear. This study aimed to determine the smallest detectable change (SDC) of the Japanese KNAP among female teleworkers with chronic neck/shoulder stiffness, a population with high levels of presenteeism.

Methodology

This test-retest reliability study was conducted alongside a randomized controlled trial involving female teleworkers with presenteeism related to neck/shoulder stiffness. Participants who completed the final one-year follow-up and who still had neck/shoulder stiffness were invited. The KNAP was administered via a web-based survey twice, with a one-week interval. The SDC was calculated using the intraclass correlation coefficient.

Results

A total of 101 participants completed two surveys. The mean KNAP total score was 75.6 (standard deviation (SD) 3.7). The SDC was 6.5.

Conclusion

The KNAP demonstrated a smallest detectable change of 6.5 points in female teleworkers with chronic neck/shoulder stiffness, indicating the threshold for changes beyond measurement error at the individual level. This finding contributes to the interpretability of the KNAP and may assist in evaluating changes over time, although the clinical importance of such changes remains to be established.

## Introduction

Pain Science Education (PSE) aims to improve patients’ understanding of pain based on modern pain science, empowering informed care choices and supporting better health outcomes [1]. PSE has been reported to be effective in reducing pain and improving outcomes in individuals with chronic musculoskeletal conditions [2-4]. To evaluate such changes, appropriate measurement tools are required to assess pain-related knowledge and attitudes [5].

The Knowledge and Attitudes of Pain (KNAP) was developed by Beetsma et al. [6] as a self-report measure to assess knowledge of modern pain science and biopsychosocial attitudes toward pain among physical therapy students in 2020. Beetsma et al. [6] examined its structural validity, internal consistency, and responsiveness among physical therapy students in the Netherlands. In 2022, a Japanese version of the KNAP was created that is comprehensible to first- and second-year undergraduate students studying physical therapy or occupational therapy who do not have specialized knowledge of pain. Therefore, the Japanese KNAP may be applicable in clinical and educational settings.

However, for any outcome measure used in repeated assessments, it is essential to understand how much change reflects true change rather than measurement error. Beetsma et al. [6] examined test-retest reliability of the KNAP among physical therapy students in the Netherlands and reported the smallest detectable change (SDC) of 4.99. However, the magnitude of measurement error and the SDC have not been sufficiently investigated in patient populations.

Chronic pain not only affects individuals but also imposes a burden on society and the economy. For example, according to the Japanese Comprehensive Survey of Living Conditions [7], women (30.4%) report being aware of symptoms of illness or injury more frequently than men (12.0%). Furthermore, among women, neck/shoulder stiffness, along with low back pain, has the highest prevalence rate at 10.6%. Moreover, neck/shoulder stiffness is not limited to Japan and has also been reported at a high prevalence rate in small-scale surveys of around 100 people conducted in other countries (61.8% in the United States and 75.1% in Singapore [8]). Neck/shoulder stiffness is included in chronic pain management guidelines in Japan [9], indicating that chronic neck/shoulder stiffness is identified as one of the most important clinical issues. Further, neck/shoulder stiffness has been identified as the second most strongly associated symptom with presenteeism, followed by mental health problems, and a reported annual productivity loss due to neck/shoulder stiffness was reported to be $414 per person per year [10]. It is also known that presenteeism is highest among female teleworkers [11]. Consequently, it is of interest to examine how comprehensive interventions, including PSE, contribute to improvements in pain-related and work-related conditions among this population. Thus, this study aimed to estimate the SDC of the KNAP in female teleworkers with chronic neck/shoulder stiffness.

## Materials and methods

Design

This study was conducted alongside a randomized clinical trial (RCT) (UMIN000054645) involving two groups of female teleworkers who reported presenteeism associated with chronic neck/shoulder stiffness. The trial, which will be reported separately, compared a group that received an educational booklet on the work environment with a group that received the same booklet, in addition to a three-month online program based on the McKenzie method of Mechanical Diagnosis and Therapy (MDT). For a test-retest investigation, individuals with stable symptoms without any active intervention are required. Therefore, this study recruited individuals from those who completed the RCT at the final one-year follow-up. This study was approved by the Saitama Prefectural University Research Ethics Committee (No. 25040). Data collection was conducted via a designated web survey following the acquisition of written informed consent. All procedures adhered to the principles outlined in the Declaration of Helsinki. Data were collected from September 2025 to March 2026.

Participants

The RCT recruited 150 female teleworkers who had experienced neck/shoulder stiffness for more than three months, felt that neck/shoulder stiffness had impaired their work performance, and whose Work Functioning Impairment Scale (7-35) was >13, indicating presenteeism [12,13]. The current study recruited participants who completed the one-year follow-up of the aforementioned RCT. Eligibility criteria included: (1) female teleworkers with neck/shoulder stiffness; (2) Japanese residents whose primary language was Japanese; and (3) full-time workers, including self-employed individuals, who mainly performed desk work and teleworked for more than 70% of the workweek. The exclusion criteria were (1) those who cannot access the internet, do not have the skills to use the internet, or do not have an environment in which video communication is available, (2) those who were pregnant or had a history of neck surgery, (3) diagnosis with any permanent musculoskeletal disorder; or diagnosis of mental, neurological, or respiratory disease including asthma, (4) those who are receiving any continuous treatment for neck/shoulder stiffness in hospitals now and in the future at the time of recruitment, excluding massage, moxibustion, and acupuncture at non-hospital institutions, (5) those who are visiting a hospital due to a musculoskeletal disorder in a region other than the neck. One week after the initial KNAP survey, participants received a link to the second web survey and were asked to complete it. Age and symptom location [14] were collected as basic participant characteristics. 

The KNAP

This study used the Japanese KNAP [15]. The KNAP consists of 30 statements regarding knowledge of modern pain science and biopsychosocial attitudes toward pain. Respondents rate their level of agreement with each statement on a six-point Likert scale. Structural validity based on exploratory factor analysis and Rasch analysis, internal consistency (Cronbach’s alpha of 0.8), hypothesis testing, test-retest reliability, and responsiveness were examined using data from physical therapy students in the Netherlands [6]. A study of Japanese participants found that, compared to general physical therapists, physical therapists with the MDT credential license provide more appropriate pain management and achieve higher KNAP scores [16]. These results suggest construct validity for the KNAP. A previous study [6] developed a Rasch-based conversion table to support unidimensional scoring of the KNAP. Using the conversion table, item responses were converted to weighted scores (four to six points), and the summed scores were subsequently transformed into Rasch-scaled total scores ranging from 0 to 150.

Analysis

IBM® SPSS® Statistics for Windows version 28.0 (IBM Corp, Armonk, NY) was used for statistical analyses. The significance level was set at 5%. Descriptive analyses were used to summarize characteristics of the participants.

Regarding the total KNAP score, the intraclass correlation coefficient (ICC) was calculated using a two-way random-effects model. Then, the standard error of measurement (SEM) was estimated as the square root of the mean square error from the F-test and SDC were calculated using the following formula:

$SDC=SEM&times;1.96&times;\sqrt{2}$

## Results

**Figure 1:**
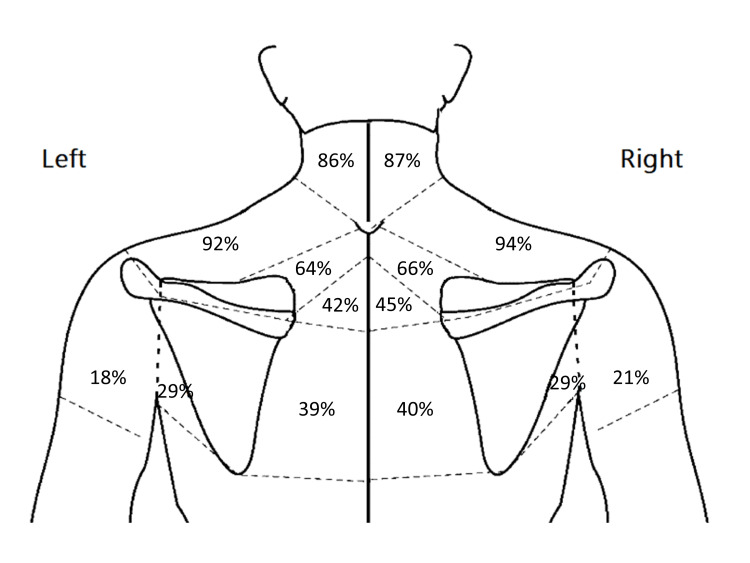
Symptom locations of the 101 participants. This figure was modified with permission from the copyright holder, the Igaku-Shoin Co., Ltd. Source: Shinozaki T, Osawa T, Tsutsumi S, Kobayashi T, Takagishi K. Pathophysiology of stiff shoulders - based on a questionnaire survey. Rinsho Seikei Geka. 2007;42(5):409-12.

In the initial survey, responses were received from 125 participants (mean (SD) age of 40.1 (10.2) years), and in the second survey, responses were received from 101 participants (mean age of 40.6 (10.1) years). There were no missing values for the KNAP score. Figure 1 shows the symptom location of the 101 participants.

**Figure 2:**
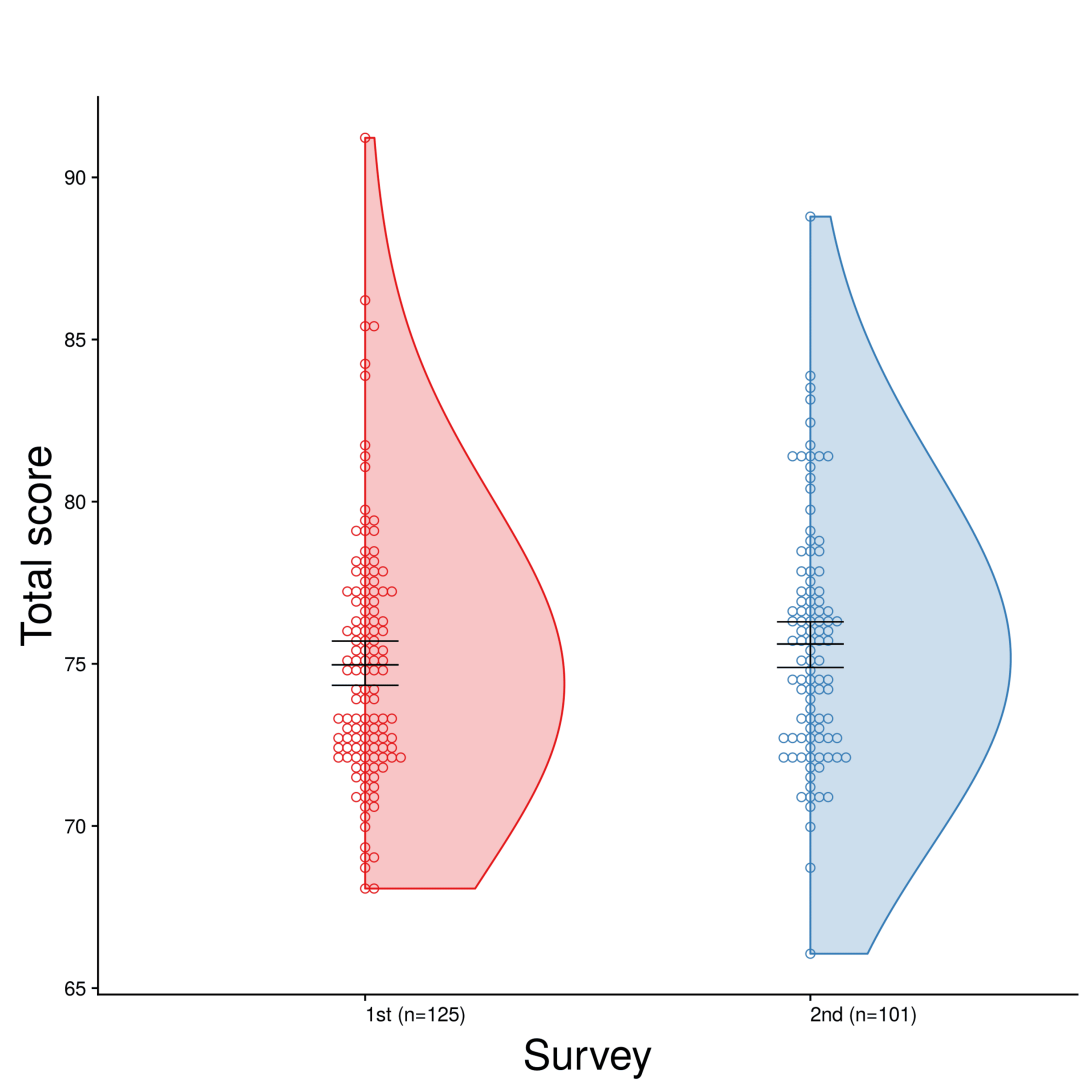
Data distributions of the Knowledge and Attitudes of Pain total score. Means and 95% confidence intervals are presented with bars. Raw data points and violin plots are presented.

The mean (SD) response interval was 7.1 (1.9) days. Figure 2 presents data distributions of the KNAP total scores. Table 1 shows the results of the statistical analysis.

**Table 1:** Results of the analysis. Abbreviation: KNAP, Knowledge and Attitudes of Pain. Values are presented as point estimates (95% confidence intervals). *n=125; ^†^n=101.

Variable	Value
Cronbach’s alpha for the first survey*	0.63 (0.54–0.72)
Cronbach’s alpha for the second survey^†^	0.62 (0.50–0.72)
Intraclass correlation coefficient for the KNAP total score^†^	0.61 (0.47–0.72)
Standard error of measurement^†^	2.3
Smallest detectable change^†^	6.5

## Discussion

This study estimated the SDC of the KNAP in a patient population, specifically female teleworkers with chronic neck/shoulder stiffness. The SDC of 6.5 points represents the threshold beyond which observed changes can be interpreted as exceeding measurement error at the 95% confidence level. These findings contribute to the interpretability of the KNAP when applied to patients, a context in which such information has been limited.

From a measurement perspective, the SDC provides a practical benchmark for distinguishing true change from measurement error at the individual level. This is particularly relevant when the KNAP is used in longitudinal assessments, where repeated measurements are intended to capture changes in pain-related knowledge and attitudes. By quantifying the magnitude of change required to exceed measurement error, the present findings may inform the interpretation of KNAP scores in similar populations.

The present study found limited test-retest reliability (intraclass correlation coefficient (ICC)=0.61) and internal consistency (Cronbach’s alpha=0.62-0.63) compared with the commonly accepted threshold of 0.7 [17]. These results suggest some limitations in the consistency of the KNAP in the current population. However, reliability indices such as the ICC reflect relative reliability, whereas the SDC reflects absolute measurement error. These properties provide complementary information. In this study, the primary aim was to estimate measurement error to support interpretability; therefore, emphasis was placed on the SDC. Nevertheless, the relatively low internal consistency observed may indicate variability in how the construct of pain-related knowledge and attitudes is represented in this population. Given that the KNAP includes items related to both knowledge and attitudes, the assumption of a single homogeneous construct may not fully hold, and further investigation using alternative reliability indices or item-level analyses may be warranted.

Compared with previous studies, the SDC observed in this study was comparable to estimates reported in a previous study involving physical therapy students in the Netherlands [6], despite differences in populations and calculation methods. This consistency may suggest that the measurement error of the KNAP is relatively stable across different groups. Further, some previous studies have interpreted SDCs of less than 10% and 10-30% of the total score as indicating excellent and acceptable precision [18,19]. When using this criterion, since the KNAP has a score range of 0-150, it is interpreted as having excellent measurement precision. However, the lower internal consistency observed in the present study compared to the previous study in the Netherlands (Cronbach’s alpha of 0.8) [6] indicates that the performance of the KNAP may vary depending on the characteristics of the target population. This highlights the importance of evaluating measurement properties in the specific population in which the instrument is intended to be used.

Several limitations should be acknowledged. First, the sample was restricted to female teleworkers with chronic neck/shoulder stiffness, who were recruited from a prior RCT related to pain management. Therefore, previous exposure to educational material may have influenced their baseline knowledge and attitudes, which limits the generalizability of the findings in addition to the limitation to the female teleworkers with chronic neck/shoulder stiffness. Second, stability between measurements in relation to pain knowledge and attitudes was assumed based on the absence of active intervention, the chronic nature of the symptoms, and a short period between the two measurements. However, stability between measurements in relation to pain knowledge and attitudes was not directly assessed using an external measure. Further, such a short interval between the measurements may allow participants to remember previous responses, potentially affecting test-retest reliability. Researchers need to take these points into account when designing future studies, and it will likely be necessary to identify MICs that are more directly applicable to clinical practice than SDCs. Finally, internal consistency was lower than commonly accepted thresholds, which may reflect heterogeneity in the construct being measured or differences in the interpretation of items in the current population.

## Conclusions

In conclusion, this study provides an estimate of the measurement error of the KNAP in a patient population of female teleworkers with chronic neck/shoulder stiffness. This study contributes to the interpretability of the KNAP by identifying an SDC of 6.5 points that may assist in interpreting repeated KNAP measurements in this population, although the clinical importance of such changes remains to be established.
